# TYRO protein tyrosine kinase-binding protein predicts favorable overall survival in osteosarcoma and correlates with antitumor immunity

**DOI:** 10.1097/MD.0000000000030878

**Published:** 2022-09-30

**Authors:** Hai-Ru Xu, Jun-Jie Chen, Jin-Ming Shen, Wei-Hang Ding, Jie Chen

**Affiliations:** a Department of Orthopaedic, The First Affiliated Hospital of Zhejiang Chinese Medical University, Hangzhou, Zhejiang, China.

**Keywords:** antitumor immunity, bioinformatics, osteosarcoma, overall survival, TYROBP

## Abstract

To explore the prognostic significance and underlying mechanism of TYRO protein tyrosine kinase-binding protein (TYROBP) in osteosarcoma. Firstly, the expression of TYROBP was analyzed using the *t* test. The Kaplan–Meier plotter analysis and a receiver operating characteristic curve were performed to evaluate the influence of TYROBP on overall survival (OS). Further, Cox regression analysis was conducted to predict the independent prognostic factors for OS of osteosarcoma patients, and a nomogram was constructed. Then, the relationship between TYROBP and clinicopathological characteristics was determined using statistical methods. Enrichment analyses were conducted to evaluate the biological functions of TYROBP. Finally, the ESTIMATE algorithm was used to assess the association of TYROBP with immune cell infiltration. TYROBP was significantly increased in osteosarcoma (all *P* < .001). However, the high expression of TYROBP was related to better OS in osteosarcoma patients. Cox regression analysis showed that TYROBP was an independent prognostic factor for predicting OS (*P* = .005), especially in patients of the male sex, age <18 years, metastasis, and tumor site leg/foot (all *P* < .05). Besides, TYROBP mRNA expression was significantly associated with the tumor site (*P* < .01) but had no remarkable relationship with age, gender, and metastasis status (all *P* > .05). Functional annotation and gene set enrichment analysis (GSEA) revealed that TYROBP was mainly involved in immune-related pathways. Importantly, TYROBP positively correlated with immune scores (*P* < .001, *R* = .87). TYROBP served as an independent prognostic biomarker for OS in osteosarcoma. High TYROBP expression might prolong the survival of osteosarcoma patients mainly through promoting antitumor immunity.

## 1. Introduction

Osteosarcoma is the most commonly diagnosed primary malignant tumor of bone in children and adolescents, which is featured with a high metastasis rate and poor prognosis.^[1,2]^ There are two major hypotheses about the cellular origin of osteosarcoma including the osteoblast origin hypothesis and the mesenchymal stem cell (MSC) origin hypothesis.^[3–5]^ The osteoblast hypothesis shows that osteosarcoma originates from defective differentiation of osteoblast-committed cells, while the MSC hypothesis suggests that a mutation-carrying MSC leads to osteosarcoma.^[6]^ Osteosarcoma often occurs in the metaphysis of long bones, with an incidence of 4.4 per million people globally.^[7]^ It is estimated that pathological fracture is the first sign in 5% to 10% of patients.^[8]^ The traditional symptoms of cancer such as fever, malaise, and weight loss are not sensitive to children.^[9]^ With the advancement of neoadjuvant chemotherapy, radiotherapy, and surgical resection, the prognosis of osteosarcoma patients has significantly improved.^[10]^ However, approximately 20% of patients with osteosarcoma would develop lung metastasis.^[11]^ The 5-year survival rate for patients with primary osteosarcoma is 60% to 70%, but it is only 20% for those with lung metastasis. Therefore, it is of urgency to find effective biomarkers for improving the diagnosis and prognosis of osteosarcoma.

TYRO protein tyrosine kinase-binding protein (TYROBP), also known as DAP12, is an encoding gene of a transmembrane signaling polypeptide and possesses an immunoreceptor tyrosine-based activation motif in its cytoplasmic domain.^[12]^ TYROBP is predominantly expressed in oligodendrocytes, osteoclasts, natural killer cells, and macrophages in peripheral organs,^[13–15]^ which modulates the functions of these immune cells through the regulation of inhibitory and activating signals.^[16,17]^ It has been reported that TYROBP was involved in the pathogenesis of late-onset Alzheimer’s disease (AD), and identified as a “driver gene” via a multiscale computational network.^[18]^ Besides, bone remodeling and brain function also depend on the integrity of TYROBP signaling.^[13]^ Wu et al demonstrated that TYROBP was significantly enriched in the immune system and might represent an essential role in the pathogenic inflammatory response of clear cell renal cell carcinoma.^[19]^ However, the role of TYROBP in osteosarcoma has not been clarified.

Using the RNA sequencing and clinical data of osteosarcoma based on therapeutically applicable research to generate effective treatments (TARGET) and gene expression omnibus (GEO), we determined the role of TYROBP in the progression of osteosarcoma by bioinformatics approaches. We observed a high expression of TYROBP in osteosarcoma and explored its effect on the osteosarcoma prognosis. Then, the relationship between TYROBP and clinicopathological factors was analyzed. In addition, enrichment analyses were carried out to obtain the TYROBP-associated pathways, and the association of TYROBP with the tumor immune microenvironment was evaluated.

## 2. Materials and Methods

### 2.1. Data mining

GDC TARGET-osteosarcoma gene expression RNA sequencing data and corresponding clinical information were downloaded from the UCSC Xena database (https://xenabrowser.net/). Patients with incomplete clinical information such as survival status, survival time, age, and gender or those without expression value were excluded, a total of 76 osteosarcoma patients were enrolled. Moreover, two publicly available datasets were obtained from the GEO database (https://www.ncbi.nlm.nih.gov/geo/). The GSE42352 dataset includes expression data for osteosarcoma, mesenchymal stem cells, and osteoblasts. GSE21257 involves expression and related clinical data of osteosarcoma.

### 2.2. Expression analysis of TYROBP

GEO is a public functional genomics data repository supporting MIAME-compliant data submissions. The GSE42352 dataset was obtained to analyze the TYROBP mRNA expression in osteosarcoma and mesenchymal stem cell groups. In addition, the mRNA expression levels of TYROBP in osteosarcoma and osteoblasts were evaluated.

### 2.3. Clinical significance of TYROBP in osteosarcoma

First, the overall survival (OS) between high and low TYROBP expression groups was evaluated by the Kaplan–Meier plotter method based on GDC TARGET-osteosarcoma data. The diagnostic value of TYROBP was assessed by calculating the area under the curve (AUC) of the receiver operating characteristic (ROC) curve. The computed AUC value ranging from 0.5 to 1.0 indicated 50% to 100% discrimination ability. Then, GSE21257 was used to verify the prognostic value of TYROBP in osteosarcoma using Kaplan–Meier plotter and ROC analyses. Following this, univariate and multivariate Cox regression analyses were carried out to determine the prognostic factors affecting OS in osteosarcoma patients. The “rms” in the R package was adopted to construct a nomogram and calibration plots to predict 3- and 5-year OS for osteosarcoma patients. The concordance index was used to analyze the predictive accuracy of the nomogram. The R package “forestplot” and multivariate Cox regression analysis were used for the clinicopathological subgroup study. Patients were divided into different groups according to gender, age, metastasis status, and tumor site.

### 2.4. The association of TYROBP expression with clinicopathological characteristics

The clinicopathological characteristics were compared between the high (50%) and low TYROBP (50%) expression groups. Moreover, the relationship between the expression of TYROBP as a continuous variable and clinical parameters was evaluated.

### 2.5. Identification and function annotation of differentially expressed genes (DEGs) in osteosarcoma

Totally 76 patients with osteosarcoma were divided into high and low TYROBP expression groups based on the median expression value of TYROBP. The R package “limma” was adopted to compare the expression data of two groups in osteosarcoma samples to identify DEGs, where the |Log2FC| >1, and *P* < .05 were set as the thresholds. Then, these DEGs were processed for functional annotation including gene ontology and Kyoto encyclopedia of genes and genomes (KEGG) pathway using the clusterProfiler in R package. *P* value < .05 and false discovery rate < 0.25 were considered statistically significant.

### 2.6. Gene set enrichment analysis (GSEA)

The R package clusterProfiler was utilized for GSEA with normalized RNA-seq data from TARGET to explore the mechanisms of TYROBP expression on the progression of osteosarcoma. The gene set was permutated 1000 times and the expression level of TYROBP was used as a phenotypic label. Clusters with a nominal *P* value < .05, and a false discovery rate *q* value < .25 were considered as significant.

### 2.7. Generation of the immune score and stromal score

Using the ESTIMATE package, we estimated the ratio of immune and stromal components in each sample in the tumor microenvironment in the form of the immune score and stromal score, which are positively related to the ratio of immune and stroma, respectively.^[20]^

### 2.8. Statistical analysis

All statistical analyses were performed by SPSS software (SPSS, Inc., Chicago, IL), and R software. The categorical data were expressed as n (%) and were analyzed using the chi-square test. The continuous data were expressed as mean ± SD, comparisons between two groups were assessed by independent sample *t* test, and comparisons among multiple groups were evaluated using a one-way analysis of variance. Low and high TYROBP expressers were discriminated against according to the median expression level of TYROBP. The Kaplan–Meier plotter method was adopted to construct the survival curves using the log-rank test. Hazard ratio (HR) and 95% confidence interval were calculated in both univariate and multivariate Cox regression analyses. The relationship between TYROBP expression and the immune score was analyzed by the Pearson Correlation test. All statistical tests were double-tailed with 0.05 as the statistical significance level.

### 2.9. Ethical statement

All data in this study were obtained from open public databases; we did not obtain these data from patients directly or intervene with these patients. Therefore, ethical approval was not required for this study.

## 3. Results

### 3.1. High TYROBP expression predicted better OS

**Figure 1.**
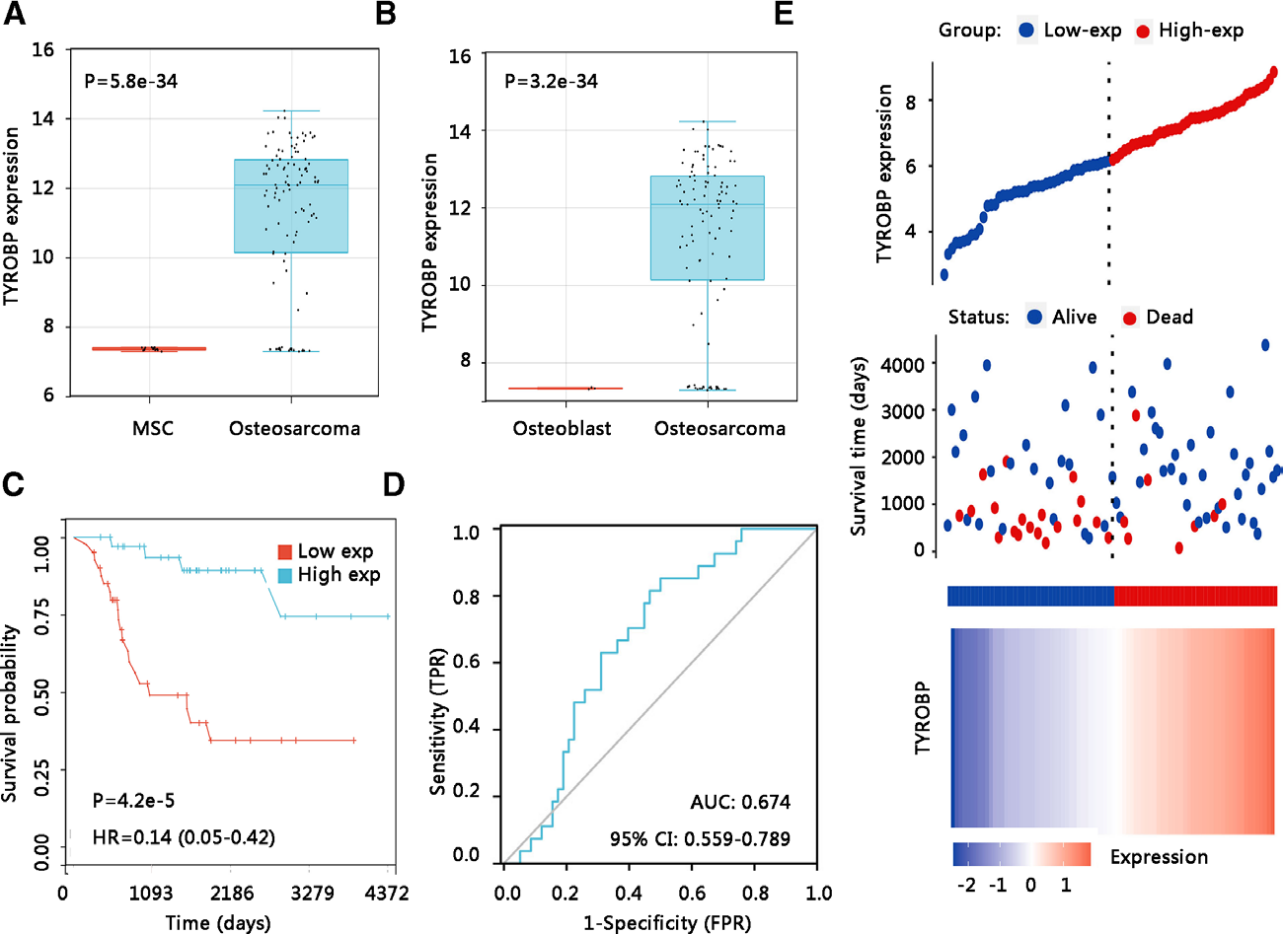
The relationship between TYROBP expression and overall survival in osteosarcoma. GSE42352 dataset: The differential expression of TYROBP in osteosarcoma patients and (A) mesenchymal stem cells and (B) Osteoblasts. GDC TARGET-osteosarcoma: (C) The effect of TYROBP on overall survival. (D) Receiver operating characteristic curve of TYROBP expression. (E) TYROBP expression distribution and survival status. AUC = area under the curve, CI = confidence interval, HR = hazard ratio, MSC = mesenchymal stem cell, TARGET = therapeutically applicable research to generate effective treatments, TYROBP = TYRO protein tyrosine kinase-binding protein.

**Figure 2.**
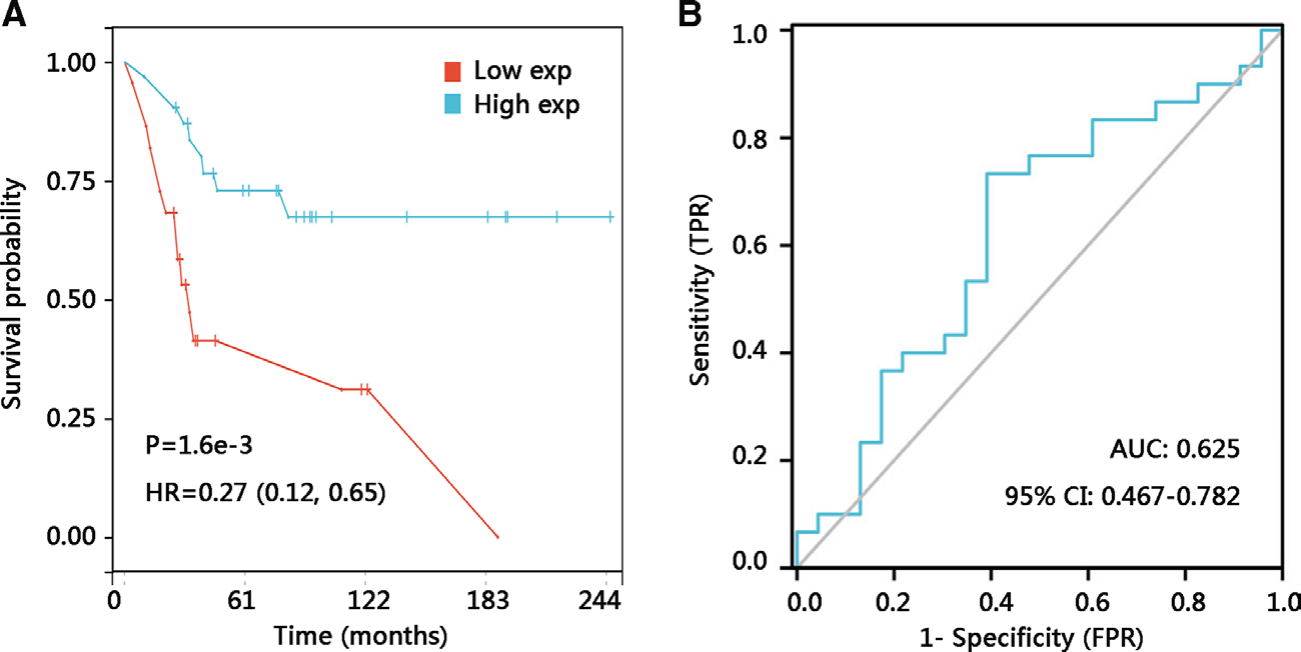
Verification of the prognostic value of TYROBP in osteosarcoma using the GSE21257 dataset. (A) Kaplan–Meier plotter curves of the impact of TYROBP on the overall survival. (B) Receiver operating characteristic curve of TYROBP expression. AUC = area under the curve, CI = confidence interval, HR = hazard ratio, TYROBP = TYRO protein tyrosine kinase-binding protein.

To explore the expression level of TYROBP in osteosarcoma, the expression data from GSE42352 was analyzed and a significantly increased expression of TYROBP was observed in the osteosarcoma group compared with the mesenchymal stem cell group (*P* < .001) (Fig. 1A). Besides, TYROBP was highly expressed in osteosarcoma than that in osteoblast (*P* < .001) (Fig. 1B). To investigate the clinical benefits of TYROBP, we performed a Kaplan–Meier plotter analysis of the GDC TARGET-osteosarcoma dataset and found that osteosarcoma patients with high TYROBP expression exhibited better OS (HR = 0.14, *P* < .001) (Fig. 1C). Next, the ROC curve was used to demonstrate its value in distinguishing the survival status of osteosarcoma. As AUC was 0.674, TYROBP showed acceptable specificity and sensitivity for discriminating the survival status (Fig. 1D). The distribution of TYROBP expression, survival status of osteosarcoma patients, and expression profiles of TYROBP were presented in Figure 1E. Further, the relationship between TYROBP expression and OS was validated using GSE21257 data. Consistent with the TARGET results, the up-regulated TYROBP expression led to a favorable OS in osteosarcoma patients (HR = 0.27, *P* < .01) (Fig. 2A). TYROBP expression presented a potential prognostic value as the ROC curve showed that the AUC of TYROBP expression for predicting survival status was 0.625 (Fig. 2B).

### 3.2. TYROBP was an independent prognostic factor in osteosarcoma

To determine the independent prognostic significance of TYROBP for OS in osteosarcoma, Cox regression analysis was performed using GDC TARGET-osteosarcoma data. Univariate regression analysis showed that non-metastasis (HR = 0.225, *P* < .001) and high TYROBP expression (HR = 0.584, *P* < .001) were significantly related to the prolonged OS time. However, age, gender, leg/foot, and pelvis had no remarkable relationship with the clinical outcomes. In multivariate regression analysis, non-metastasis (HR = 0.245, *P* = .003) and high TYROBP expression (HR = 0.629, *P* = .005) were still independent factors for favorable OS in patients with osteosarcoma (Table 1).

**Table 1:** Cox regression analysis of TYROBP expression and overall survival for patients with osteosarcoma.

Characteristics	Univariate analysis		Multivariate analysis	
	HR (95 % CI)	*P* value	HR (95 % CI)	*P* value
Age	1.005 (0.921–1.097)	.908	1.057 (0.957–1.167)	.272
Gender (male vs female)	0.775 (0.346–1.737)	.535	0.699 (0.276–1.773)	.451
Metastasis status (M0 vs M1)	0.225 (0.100–0.505)	<.001	0.245 (0.098–0.612)	.003
Leg/foot	0.963 (0.129–7.187)	.971	1.585 (0.178–14.155)	.680
Pelvis	4.606 (0.413–51.309)	.214	7.989 (0.590–108.217)	.118
TYROBP	0.584 (0.436–0.781)	<.001	0.629 (0.456–0.868)	.005

CI = confidence interval, HR = hazard ratio, M0 = no metastasis, M1 = metastasis, TYROBP = TYRO protein tyrosine kinase-binding protein.

**Figure 3.**
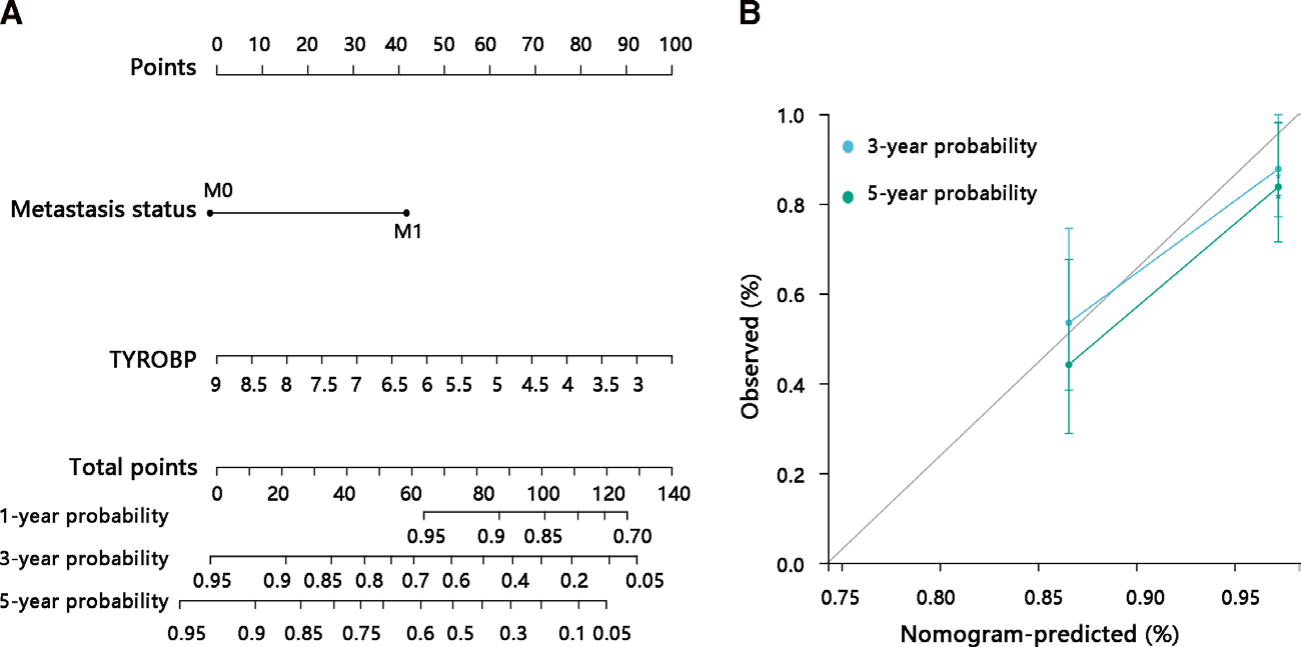
Construction and validation of a nomogram based on TYROBP. (A) Nomogram for predicting the probability of 3-, and 5-year overall survival for osteosarcoma. (B) Calibration curve to assess the accuracy of the nomogram. M0 = no metastasis, M1 = metastasis.

To better predict the prognosis of osteosarcoma patients, a nomogram based on the Cox regression analysis results was constructed and a calibration curve was plotted to evaluate the efficiency of the nomogram. Two statistically significant prognostic factors metastasis status and TYROBP expression were included in the model to predict the OS, which had a concordance index of 0.774 (Fig. 3A). The calibration curve presented a desirable prediction of the nomogram for the 3-, and 5-year survival probability (Fig. 3B).

### 3.3. Prognostic significance of TYROBP in the osteosarcoma subgroups

**Figure 4.**
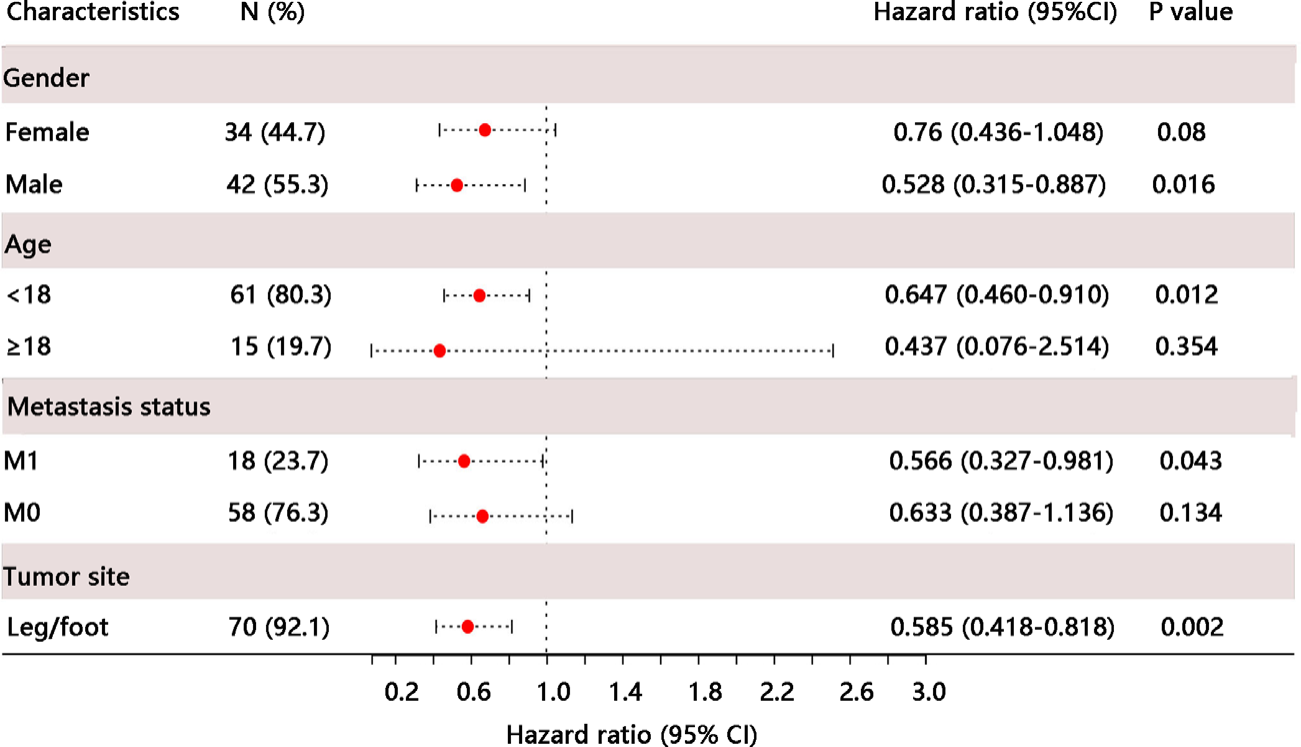
Prognostic performance of TYROBP on overall survival in osteosarcoma patient subgroups. TYROBP = TYRO protein tyrosine kinase-binding protein.

**Figure 5.**
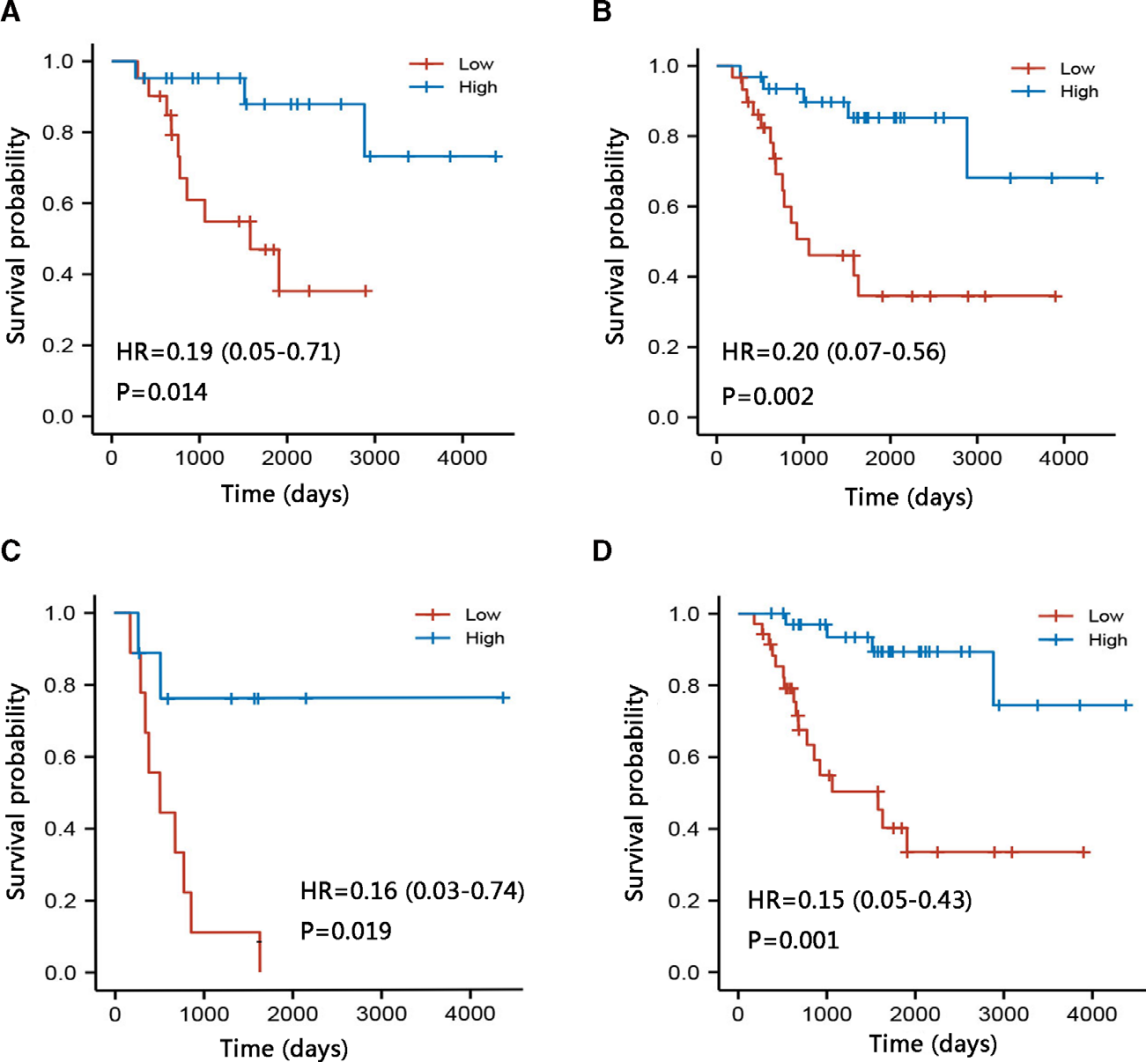
Effect of TYROBP expression on overall survival in osteosarcoma patient subgroups using Kaplan–Meier plotter. (A) Male. (B) Age below 18 years. (C) Metastasis. (D) Leg/foot. TYROBP = TYRO protein tyrosine kinase-binding protein.

Subsequently, we examined the prognostic value of TYROBP for OS in certain clinicopathological subgroups. Multivariate Cox regression analysis was conducted in specific subgroups. As shown in Table 2, TYROBP was an independent prognostic factor for OS in patients of male sex (HR = 0.528, *P* = .016), age below 18 years (HR = 0.647, *P* = .012), metastasis (HR = 0.566, *P* = .043), and tumor site at leg/foot (HR = 0.585, *P* = .002). Likewise, the forest plot illustrated the independent prognostic value of TYROBP in osteosarcoma with restricted characteristics using multivariate Cox regression results (Fig. 4). The subgroup analyses for tumor site (arm/hand, and pelvis) were not performed due to few samples. The Kaplan–Meier plotter analysis for OS was performed in these four subgroups: male, age below 18 years, metastasis, and tumor site leg/foot (all *P* < .05) (Fig. 5A–D). the high TYROBP expression groups.

**Table 2:** Prognostic performance of TYROBP on overall survival in osteosarcoma patient subgroups by multivariate Cox regression analysis.

Characteristics	N (%)	Hazard ratio (95%CI)	*P* value
Gender			
Female	34 (44.7)	0.76 (0.436–1.048)	.08
Male	42 (55.3)	0.528 (0.315–0.887)	.016
Age			
<18	61 (80.3)	0.647 (0.460–0.910)	.012
≥18	15 (19.7)	0.437 (0.076–2.514)	.437
Metastasis status			
M1	18 (23.7)	0.566 (0.327–0.981)	.043
M0	58 (76.3)	0.663 (0.387–1.136)	.134
Tumor site			
Leg/foot	70 (92.1)	0.585 (0.418–0.818)	.002

### 3.4. Correlation between TYROBP expression and clinical characteristics

Since high TYROBP mRNA expression was closely associated with favorable OS, we next explored the clinicopathological factors that could affect its mRNA expression using GDC TARGET-osteosarcoma data. As shown in Table 3, patients in the high TYROBP expression group manifested a higher proportion of leg/foot primary tumor sites than those in the low TYROBP expression group (*P* < .05). Whereas, there was no significant difference in the distribution of age, gender, or metastasis between the two groups (all *P* > .05).

**Table 3:** Relationship between TYROBP levels and clinicopathological parameters of osteosarcoma.

Variables	TYBOBP		χ^2^	*P* value
	Low (%)	High (%)		
Age (yr)			0.083	.773
<18	31 (50.8)	30 (49.2%)		
≥18	7 (46.7)	8 (53.3%)		
Gender			0.213	.645
Female	18 (52.9)	16 (47.1%)		
Male	20 (47.6)	22 (52.4%)		
Metastasis status			2.621	.105
Metastasis	12 (66.7)	6 (33.3%)		
Non-metastasis	26 (44.8)	32 (55.2%)		
Primary tumor site			8.833	.012
Arm/hand	4 (100.0)	0 (0.0%)		
Leg/foot	32 (45.7)	38 (54.3%)		
Pelvis	2 (100.0)	0 (0.0%)		

TYROBP = TYRO protein tyrosine kinase-binding protein.

**Figure 6.**
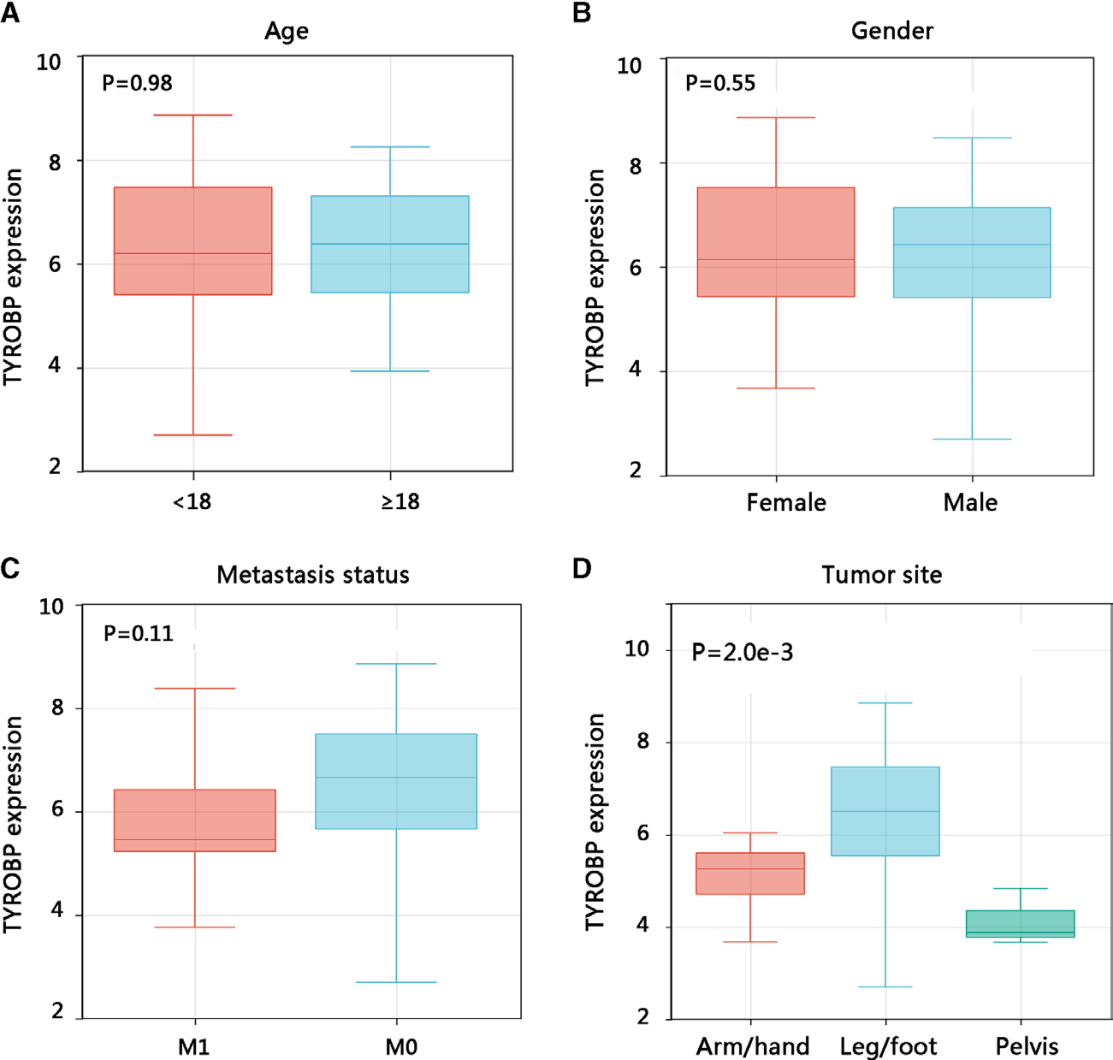
The association of TYROBP expression with clinicopathological characteristics in osteosarcoma. (A) Age. (B) Gender. (C) Metastasis status. (D) Tumor site. TYROBP = TYRO protein tyrosine kinase-binding protein.

Then, we examined the TYROBP expression in osteosarcoma patients with various clinicopathological parameters. Patients in different groups of age, gender, and metastasis shared similar TYROBP mRNA expression levels (all *P* > .05) (Fig. 6A–C), while leg/foot exhibited the highest TYROBP mRNA expression compared with other primary tumor sites with a statistical difference (*P* < .01) (Fig. 6D).

### 3.5. TYROBP regulated the progression of osteosarcoma mainly through immune-related pathways

**Figure 7.**
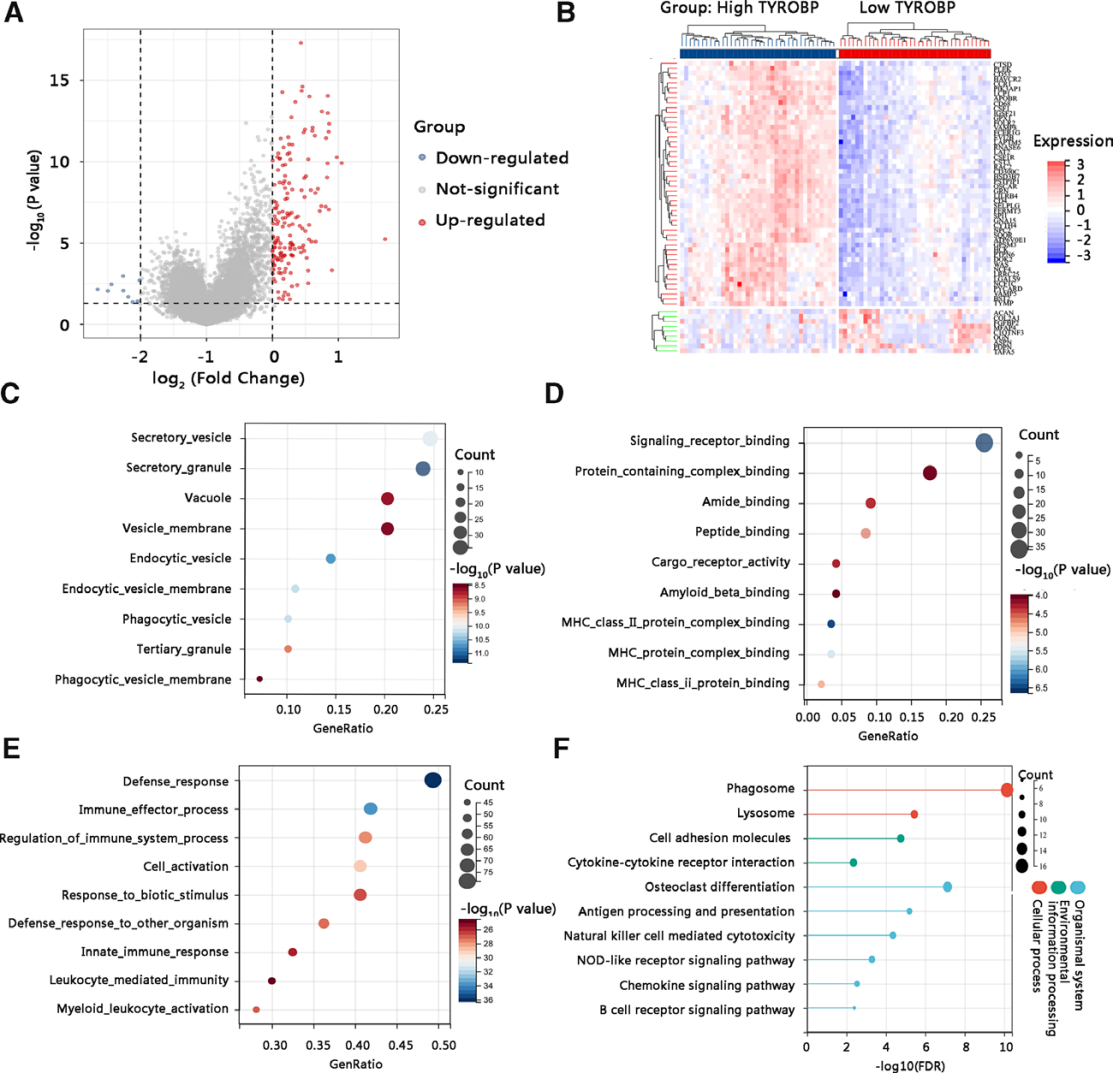
Identification and functional enrichment analysis of TYROBP-associated differentially expressed genes (DEGs) in osteosarcoma. (A) The volcano plot of the DEGs. (B) The heat map of the top 50 DEGs. (C) Cellular component. (D) Molecular function. (E) Biological process. (F) KEGG pathway. KEGG = Kyoto encyclopedia of genes and genomes, TYROBP = TYRO protein tyrosine kinase-binding protein.

To elucidate the pathological function of TYROBP in osteosarcoma, we performed gene ontology annotation and KEGG pathway analyses of DEGs between high and low TYROBP expression groups. Through differential expression analysis, we obtained 175 DEGs including 166 upregulated and 9 downregulated genes as shown in the volcano plot (Fig. 7A). The heat map presented the top 50 DEGs (Fig. 7B). In terms of the cellular components, the DEGs were mainly enriched in a secretory vesicle, secretory granule, and vacuole (Fig. 7C). The major molecular functions were signaling receptor binding, protein-containing complex binding, and amide binding (Fig. 7D). For biological processes, they were mainly involved in defense response, immune effector process, and regulation of immune system process (Fig. 7E). The KEGG pathways in which they mainly participated were cell adhesion molecules, cytokine-cytokine receptor interaction, osteoclast differentiation, antigen processing and presentation, natural killer cell-mediated cytotoxicity, NOD-like receptor signaling pathway, and chemokine signaling pathway, most of which are known to contribute to antitumor immunity (Fig. 7F).

**Figure 8.**
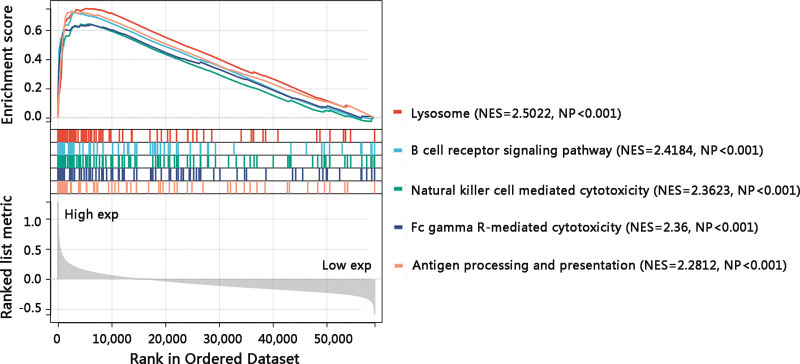
Five most significant pathways in high TYROBP expression phenotype in gene set enrichment analysis (GSEA). TYROBP = TYRO protein tyrosine kinase-binding protein.

Further, GSEA was conducted to reveal the underlying mechanism of TYROBP in osteosarcoma. The results identified that the biological pathways enriched in TYROBP high expression phenotype were lysosome, B cell receptor signaling pathway, natural killer cell-mediated cytotoxicity, Fc gamma R-mediated cytotoxicity, and antigen processing and presentation (Fig. 8). Taken together, TYROBP might affect the OS of osteosarcoma patients by regulating the antitumor immune-related pathways.

### 3.6. Association of TYROBP with the immune cell infiltrates

**Figure 9.**
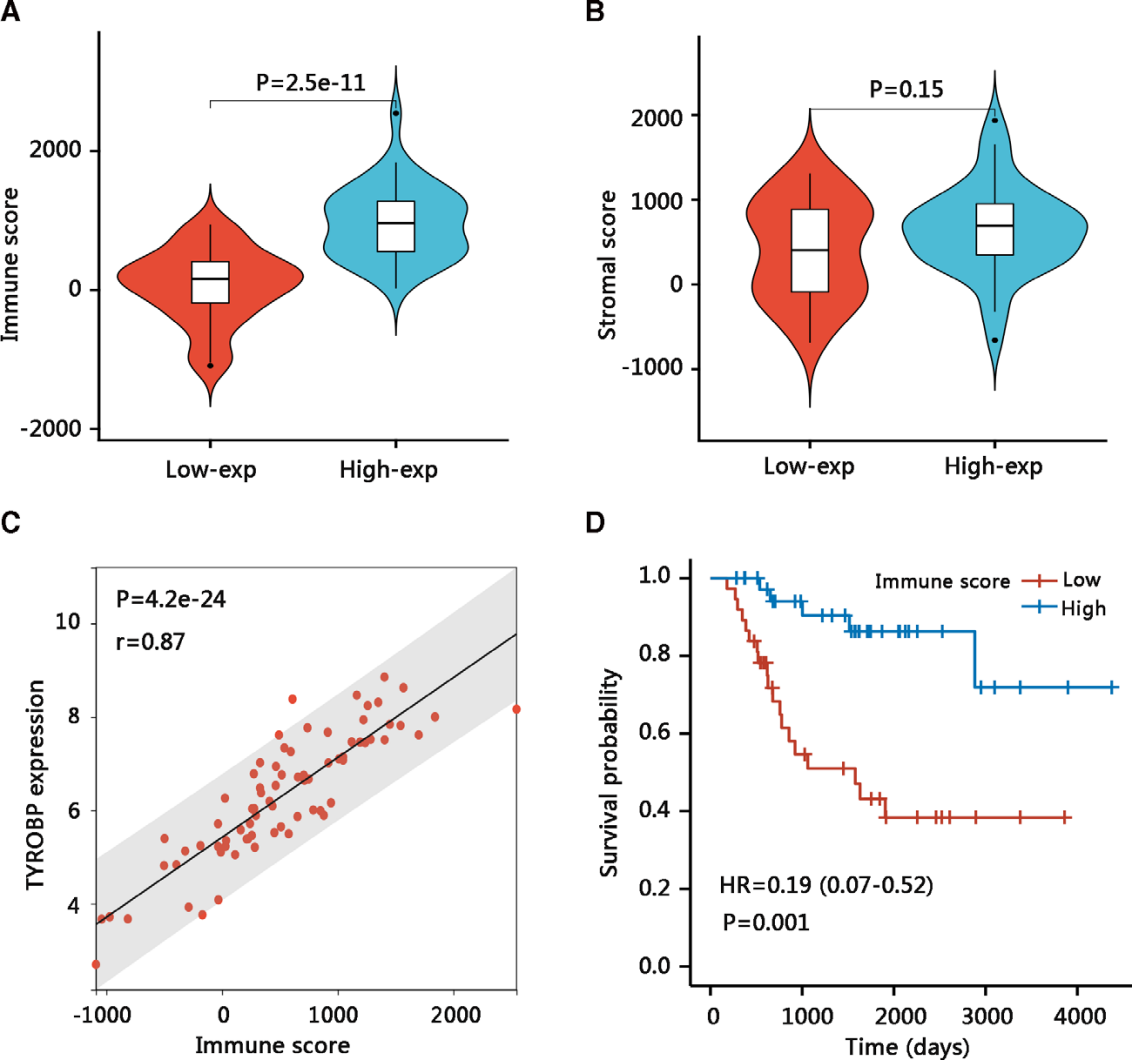
The relationship between TYROBP and tumor immune microenvironment. The relationship between TYROBP and (A) immune score and (B) stromal score. (C) Pearson correlation analysis of TYROBP expression with the immune score. (D) The effect of the immune score on the overall survival of patients with osteosarcoma. TYROBP = TYRO protein tyrosine kinase-binding protein.

We have demonstrated that TYROBP was mainly involved in immune-related pathways, and hence we attempted to explore the correlation between TYROBP and immune cell infiltration levels using the ESTIMATE algorithm. As shown in Figure 9A, the high TYROBP expression group had a significantly higher immune score, indicating a higher proportion of immune cell infiltrates (*P* < .001). Besides, the stromal score in the high TYROBP expression group was not significantly different from that in the low TYROBP expression group (Fig. 9B). Correlation analysis showed that TYROBP had a strong positive relation with immune score (*P* < .001, *R* = .87) (Fig. 9C). In addition, patients with high immune scores had longer OS (*P* < .01) (Fig. 9D). These results confirmed that TYROBP might improve the clinical outcomes of osteosarcoma patients via positive regulation of the antitumor immunity.

## 4. Discussion

As a downstream adaptor of numerous immune receptors, TYROBP is mainly expressed on the surface of the myeloid and natural killer cells, T cells, and B cells. It is also expressed on microglial cells in the brain and osteoclasts in the bone marrow, which are vital for myelination in the brain and bone resorption.^[21–23]^ Previous studies have proved that TYROBP played a critical role in various diseases. The missense mutations in the coding region of the TYROBP were closely related to the Alzheimer’s disease risk.^[24]^ Besides, the clear cell renal cell carcinoma tissues had a significantly higher TYROBP expression than the normal tissues, and its overexpression led to a lower survival probability.^[25]^ The elevated level of TYROBP was observed in nearly 66% of the breast cancers, which was related to the worse prognosis of breast cancer patients.^[26]^ Surprisingly, our study found that TYROBP was upregulated in osteosarcoma, and high expression of TYROBP contributed to the favorable OS in osteosarcoma. The ROC curve for TYROBP discrimination of survival status had an AUC of 0.674 and 0.625 based on TARGET-osteosarcoma and GSE21257 data, respectively, indicating that TYROBP was a reliable biomarker for distinguishing the survival status of osteosarcoma patients. Furthermore, TYROBP exhibited satisfactory performance on OS of osteosarcoma patients in accordance with the Cox regression analyses and nomogram construction. More importantly, high TYROBP expression served as an independent prognostic biomarker for favorable OS in osteosarcoma patients particularly in patients of the male sex, age below 18 years, metastasis, and tumor site leg/foot groups. These findings revealed that TYROBP played a diverse role in various diseases. Moreover, we demonstrated that TYROBP expression was significantly related to the tumor site, but had no remarkable relationship with age, gender, and metastasis status.

Next, we embarked on the underlying mechanisms of TYROBP in osteosarcoma. The functional enrichment analysis of TYROBP-associated DEGs showed that they were mainly enriched in osteoclast differentiation and immune-related pathways such as cell adhesion molecules, cytokine-cytokine receptor interaction, antigen processing and presentation, natural killer cell-mediated cytotoxicity, and chemokine signaling pathway. According to GSEA results, lysosome, B cell receptor signaling pathway, natural killer cell-mediated cytotoxicity, Fc gamma R-mediated cytotoxicity, and antigen processing and presentation were enriched in high TYROBP expression phenotype. Natural killer cells are lymphocytes of the innate immune system, which can recognize and eliminate allogeneic cells, tumor cells, and microbe-infected cells without prior sensitization.^[27]^ Besides, TYROBP has been demonstrated to activate natural killer cells and the TYROBP-dependent natural killer cells activating receptor NKG2D was essential in the anti-tumor process.^[28–30]^ Additionally, cancer immunotherapy relies on an appropriate target antigen and antigen presentation to the patient’s immune system.^[31]^ The chemokine signaling pathways consist of many chemokine proteins, which play a regulatory role in the recruitment of immune cells during inflammatory responses.^[32]^ Therefore, it is reasonable to speculate that TYROBP might improve the OS of osteosarcoma patients by regulating these antitumor immune-related pathways. Tumor-infiltrating immune cells are linked to angiogenesis, tumor occurrence, tumor cell growth, and metastasis.^[33]^ A higher proportion of antitumor immune cells in the high CXCL11 expression group, improving the prognosis of colon adenocarcinoma patients.^[34]^ Similarly, through the correlation analysis of TYROBP and tumor-infiltrating immune cells, a strong positive relationship between TYROB and immune scores was observed. Notably, the high immune scores group had a longer OS time than the low immune scores group, suggesting that the high TYROBP expression group might present more antitumor immune cells and hence improve the clinical outcomes of osteosarcoma patients. On the other hand, the balance of existing bone resorption by osteoclasts and new bone formation by osteoblasts is disturbed in the progression of primary or metastatic bone tumors.^[35]^ In osteoblastic tumor lesions, an excess of bone is deposited and osteoclasts are essential in the formation of these lesions.^[36,37]^ In most cases, osteosarcoma is a mixed osteoblastic and osteolytic lesion since being of the osteoblast cell lineage, osteosarcoma forms bone and promotes osteoclastogenesis. The osteoclast-targeted therapy is a potential method to address the development of osteosarcoma.^[35]^ Thus, the involvement of TYROBP in the osteoclast differentiation pathway might partially explain that high TYROBP expression contributed to better OS of osteosarcoma patients. However, the underlying mechanisms of TYROBP in osteosarcoma require further in vitro and in vivo studies in the future, and these findings should be verified in a larger cohort.

In conclusion, TYROBP was highly expressed in the osteosarcoma group and it might serve as a potential biomarker for improving the diagnosis and prognosis of osteosarcoma patients. Besides, high TYROBP expression might prolong the OS of patients with osteosarcoma by regulating the antitumor immune-related pathways and osteoclast differentiation.

## Author contributions

**Conceptualization:** Hai-Ru Xu, Jun-Jie Chen.

**Data curation:** Hai-Ru Xu, Jun-Jie Chen.

**Formal analysis:** Hai-Ru Xu, Jie Chen.

**Investigation:** Jun-Jie Chen, Jie Chen.

**Methodology:** Hai-Ru Xu, Jun-Jie Chen, Jin-Ming Shen, Wei-Hang Ding.

**Resources:** Jin-Ming Shen.

**Software:** Jin-Ming Shen, Wei-Hang Ding.

**Supervision:** Jin-Ming Shen, Wei-Hang Ding, Jie Chen.

**Validation:** Jin-Ming Shen, Wei-Hang Ding, Jie Chen.

**Visualization:** Wei-Hang Ding, Jie Chen.

**Writing – original draft:** Hai-Ru Xu, Jun-Jie Chen, Wei-Hang Ding, Jie Chen.

**Writing – review & editing:** Hai-Ru Xu, Jun-Jie Chen, Jin-Ming Shen, Jie Chen.
